# Consumer Labels can Convey Polyphenolic Content: Implications for Public Health

**DOI:** 10.1080/10446670410001722249

**Published:** 2005-03

**Authors:** Andrew L. Waterhouse

**Affiliations:** Department of Viticulture and Enology University of California One Shields Ave. Davis CA 95616-8749 USA

## Abstract

Polyphenolics are a large group of related substances. Many of these, in fact much of that found in food, is composed of processing-derived substances too complex for complete identification. Recent studies have suggested likely benefits for diets high in polyphenols, particular in reducing heart disease mortality, but other benefits have also been suggested. A consumer label based on the major polyphenolic classes is both manageable and fairly informative as most foods do not contain all possible classes. Differences between class member can be significant, but data on individual substances is impractical and no data is certainly less informative. Equivalency scales may be useful but may skew content of many foods towards the high-equivalency substances, even while the full beneficial effects of each individual substance is poorly described.


*Clinical & Developmental Immunology*, March 2005; 12(1): 43–46

### Consumer labels can convey polyphenolic content: Implications for public health

ANDREW L. WATERHOUSE

*Department of Viticulture and Enology, University of California, One Shields Ave., Davis, CA 95616-8749, USA*

**Abstract**
Polyphenolics are a large group of related substances. Many of these, in fact much of that found in food, is composed of processing-derived substances too complex for complete identification. Recent studies have suggested likely benefits for diets high in polyphenols, particular in reducing heart disease mortality, but other benefits have also been suggested. A consumer label based on the major polyphenolic classes is both manageable and fairly informative as most foods do not contain all possible classes. Differences between class member can be significant, but data on individual substances is impractical and no data is certainly less informative. Equivalency scales may be useful but may skew content of many foods towards the high-equivalency substances, even while the full beneficial effects of each individual substance is poorly described.

**Keywords:** *Polyphenolics, flavonoids, hydroxycinnamates, diet, processing*

### Introduction

Polyphenolics comprise a very large group of substances, including about 5000 characterized flavonoids, but also a very large number, probably in the tens of thousands, of uncharacterized substances. The daunting number, their close similarity, and their intractable property of binding to most other biological macromolecules makes them a most difficult chemical subject to study. Wine chemists have been coping with these issues, along with tea and wood lignin chemists for a long time. Wine is distinguished by several features. First, it is a reasonably clean matrix, mostly free of other macromolecules, such as protein and polysaccharides. Second, it contains a wide range of different classes of polyphenolics, from hydroxycinnamates to anthocyanins. Third, the chemistry of the polyphenolics has great significance to red and white wine, in particular to the taste of red wines. Consequently, there has been a long-standing interest to understand their chemistry.

The purpose of this article is to discuss some of the key issues in describing wine polyphenolics, and to consider how these issues may affect an understanding of phenolics that could be useful for informative labeling in the future.

### Discusson

### *Polyphenolics in grapes and wine*

The polyphenolics in wine come nearly all from the grape, see Figure 1. The others are trace volatile components as well as a few minor yeast fermentation products. Grape juice contains the hydroxycinnamates, so both red and white wine (made solely from juice) contain this class. The other classes are found in the skins and seeds, and so are abundant in red wine with traces in white. Grape is an unusual fruit in that they contain the tartrate esters of the simple hydroxycinnamic acids, so the presence of these substances in fruit juices is considered clear evidence of grape juice. Caftaric acid is the most abundant hydroxycinnamate in grapes.

A relatively minor but significant class of polyphenolics are the stibenes, in particular resveratrol. In grape some of these are found in this aglycone form, but most are found as glucosides with the name piceid. Light stimulates *cis*/*trans* isomerization, so in wine there are usually four substances, the two resveratrols and the two piceids. The stilbenes have attracted much attention because of their biological effects on cancer mechanisms as well as other properties (Jang 1997), and recent

Correspondence: A. L. Waterhouse, Department of Viticulture and Enology, University of California, One Shields Ave., Davis, CA 95616-8749, USA. Tel: 1 530 752 4777. Fax: 1 530 752 0382. E-mail: alwaterhouse@ucdavis.edu

ISSN 1740-2522 print/ISSN 1740-2530 online © 2005 Taylor & Francis Group Ltd.
DOI: 10.1080/10446670410001722249

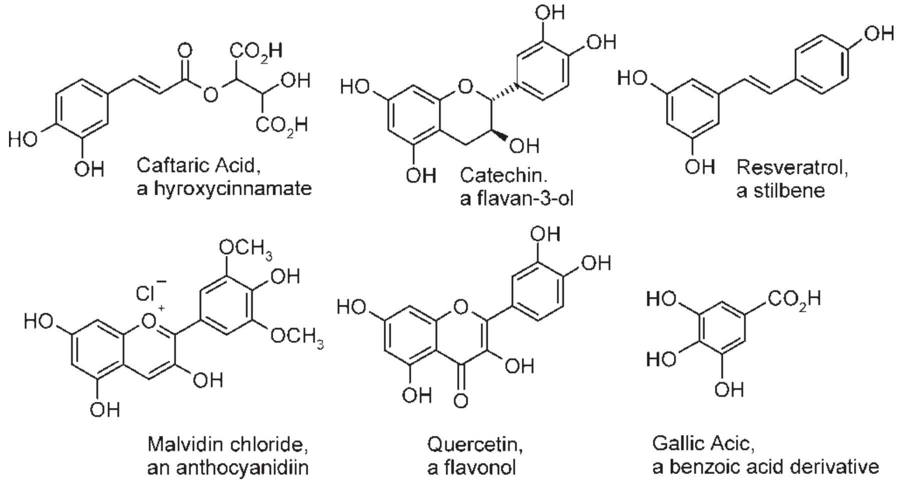

Figure 1. Representative simple flavonoids and non-flavonoids found in wine.

studies on resveratrol number in the hundreds. One last non-flavonoid class is the benzoic acids, mostly gallic acid. This is found only as an ester of other phenolics in grapes, while levels of free gallic acid are very low in new wine, gallic acid is abundant in aged red wine.

There are three major classes of flavonoids, the flavonols, anthocyanins and flavan-3-ols. The flavonols, such as quercetin, are found in the berry skin, mostly as glycosides, and during wine production and aging the glycosides slowly hydrolyze. The flavonols appear to be produced in response to sunlight (Price et al. 1995), and since at least moderate amounts of sunlight appear to be needed for high wine quality, the level of flavonols has been proposed as an indirect marker of red wine quality (Ritchey and Waterhouse 1999).

The anthocyanins are the red-colored substances found in red or black grape skin. Only a few dyer, or teinturier, varieties have red-colored juice. The anthocyanins have a very complex chemistry. In must and wine there are at least three principal forms, and only the flavylium form (than normally shown in figures, as in the case of Figure 1), about 10% of the total, is colored red. The colorless pseudo-base constitutes most of the balance and there is a small amount of quinoidal form that is purple in color. Lower pH shifts the equilibrium towards the red flavylium form, making wine darker.

Anthocyanins also react with tannins in complex and probably in multiple pathways to yield pigmented tannins (also called polymeric pigments or polymeric anthocyanins). This reaction occurs at varying rates, but continues throughout the fermentation and aging time so that in older wines, (3–10 years) all the monomeric anthocyanins are gone and the persistent color is due solely to these pigmented tannins. The specific chemical structure of these pigmented tannins is under active investigation in several labs and some recent work has suggested a few specific types of products (Waterhouse and Kennedy 2004).

The flavan-3-ols constitute the most abundant class of polyphenolics in red wine. These include the monomeric catechins, mostly catechin and epicatechin, but the vast majority exists as the series of proanthocyanidins and condensed tannins. As a group these constitute about half the polyphenolics in red wine. The size, based on the number of “catechin” subunits (degree of polymerization or DP), ranges from two to about 15, though data on DP $> 11$ is somewhat ambiguous. These are oligomers and polymers composed of subunits of catechin, epicatechin, epicatechin gallate and epigallocatechin. The inter-unit linkages have two different forms, each of which has two possible stereoisomers. Because of the diversity of subunits and linkages the number of different molecules has not been fully quantified except for DP $= 2$. The maximum number of possibilities for a ten-unit decamer has been estimated at 30 million.

There are a few chromatographic methods that separate these substances based on size or degree of polymerization. One method is based on normal phase silica gel adsorption (Rigaud et al. 1993), which works fairly well with seed tannin. Gel permeation chromatography can also be applied to the size separation of these tannins and a recent report demonstrates its utility (Kennedy and Taylor 2003).

### *Changes during fruit maturation and processing*

A key issue in understanding how these substance affect the taste of wine is to discover how they change during the maturation of the grape as well as during aging. Winegrowers state that riper fruit yields a wine with different character to the astringency. Wine drinkers also know that aging red wine results in a wine with less astringency. Once the proanthocyanidins are in wine, the linkage is susceptible to acid catalyzed cleavage followed by reaction to form a new bond (Lea 1978). Thus, the actual population of molecules is dynamic in wine and quantification usually involves measuring the amount of the class as a whole.

Changes of grape seed tannin during maturation were recently studied (Kennedy 2000) and the changes observed showed that the amount of extractable tannin decreased as the grapes ripened. Using the normal phase chromatography method mentioned above, the proportions of small and medium-sized molecules decreases, but the amount of very large polymer remained the same. So, on average, the apparent size of the extracted material increased.

In the extractable tannin, the amount that could be depolymerized by acid also decreased. This depolymerization process releases the constitutive subunits that are then trapped by a nucleophilic reagent (Kennedy and Jones 2001). In addition, this same analysis process allows the determination of polymer length because the terminal units are quantified separately. Thus, the average DP can easily be calculated from the ratio of extension units versus terminal units. The average DP is observed to decrease during ripening.

So, these two methods gave contradictory results. One showed increasing size and the other showed decreasing degree of polymerization. The conclusion was that in the seeds, the proanthocyanidins are being oxidized and subsequently cross-link in such a way that the molecule increases in size but is resistant to acid-catalyzed depolymerization. This process should also result in very large molecules or cross linking to other substrates, such as polysaccharides and subsequently prevent dissolution during extraction.

The other important consequence of this transformation is that the structure of the products is even more obscure than before. Even the basic linkage is not known. If oxidation is causing new chemical bonds to form, one is led to suspect that those linkages are more diverse than the conventional proanthocyanidin type. These substances should have potent metal binding properties as well as reactive oxygen trapping properties, and these properties could be important implication for intestinal protection (Lodovici et al. 2000).

The type of oxidation reactions described above are expected to occur during wine processing, because the wine handling that occurs in barrel maintenance in a cellar, primarily racking, does allow for oxygen incorporation. Also, a new wine treatment scheme micro-oxygenation, has been developed to increase the oxygen exposure of wine during its first year in the winery (Rieger 2000).

Tea and cocoa are also produced with oxidative treatment. The oxidative reaction products of the catechins in tea produced during the transformation to black tea are well described for the theaflavins, while the large amount of the related thearubigens are not chemically distinct (Wiseman et al. 2001). Cocoa procyanidins are deliberately oxidized during "fermentation" as well as later processing with heat and air as well as in alkalization (Bonvehi and Coll 2002). The transformation to dark brown color in both products is simple evidence of the oxidation and polymerization of flavan-3-ols often described as browning.

So, starting with fresh products that have fairly well characterized polyphenols, processing can introduce a diversity of complex new substance, most of which are not specifically known, but their general character is thought to involve oxidation-initiated bonds. They appear to have very high molecular weights and are resistant to normal analytical tools. With such diversity, it would be surprising if specific enzymes or receptors would selectively interact with these substances. On the other hand, as they are high molecular weight polyphenols, they would have potent non-specific inhibitory effects on any enzyme, complex metals very effectively, bind well to many substances, and quench reactive oxygen species very efficiently. Analyses of foods for polyphenolics could not ignore this class of substances, but they may have to be grouped into one large category.

### *Informative labeling for polyphenols*

If polyphenols are in fact one of the key food components that reduce the incidence of chronic diseases, then it is important to provide consumers with information regarding their content in foods. This should have two effects. First, it will help motivate consumers to purchase foods that contain such substances, and second, it will motivate food processors to retain these substances. Either case does not now occur because there is no means for consumers to know the content even if they wanted to find it in their food, and thus there is little demand on producers to retain it.

How could foods be labeled for polyphenols? The conventional labeling scheme involves describing the amount of particular substances considered important nutrients, such as vitamin C. The amount is determined by an analysis for ascorbate. The amount is then related to an amount recommended for good nutrition, currently called the DRI or daily recommended intake. This amount is based on a number of factors, such as the amount needed to prevent diseases of nutritional deficiency.

There are approximately 5000 described flavonoids and there are at least a few hundred non-flavonoid phenolics. Labeling that involves listing each substance is clearly impractical, but what about labeling by phenolic class? There are seven major classes of phenolics in foods: hydroxycinnamates, flavan-3-ols (or catechins), anthocyanins, flavonols, flavanonols, two classes of tannins including oligomers, and lignins. And, as described above, there is the large quantity of material that is produced by processing that is difficult to characterize. Could this be the basis of a labeling scheme?

Seven groups seems like a lot but many labels now describe many more compounds than that. In fact most foods will have just one or two flavonoid classes present, and so that will greatly simplify the label information. For instance, citrus contains only the flavanonols, and in practical terms, no other foods do. Tea contains only the flavan-3-ols. For ready differentiation from flavonols, the term catechins would be greatly preferred over flavan-3-ols for a label.

There are issues with these large groupings. For instance, the flavonol glucosides are absorbed much more efficiently than the aglycones (Hollman et al. 1995), but most foods contain mostly the glycosides. Among the flavan-3-ols, the gallated catechins have much more potent antioxidant activity than others (Liao et al. 1995). One possible means to address the latter issue is to have equivalent values for each substance, such as the system used for the tocopherols and folate. On the other hand, this skews the market to prefer only the highest potency substance, ignoring the other. Since there certainly is as yet unknown activity for many of these compounds, it would be best to simply provide an incentive to preserve the "natural" composition already present in any particular food, rather than an incentive to change it.

A label could look something like Figure 2. With only a few specific classes in each food, it would be understandable and informative. Foods that are well known for a particular class, such as teas, could be easily compared with each other. Comparison between types of foods would be less informative because of differences in the components in each class, but, no labeling scheme can be perfectly informative and also useful.

But doing nothing is riskier at least for consumers. No consumer information means that there is nothing motivating consumption or processing to preserve these substances. At present, consumer information is derived from advertisements, health magazine and similar articles. The former are motivated by a desire to sell a product, and the latter by a need to sell publications. While dangerously wrong information is probably rare, there is not recognized authority of unbiased information for consumers. Of course the published literature is "available", but the ability of consumers to find research articles and then understand them is very limited.

| | | |
|---|---|---|
| **Total Carbohydrate** 49g | 16% | 18% |
| Dietary Fiber 8g | 30% | 30% |
| Sugars 12g | | |
| Other Carbohydrate 29g | | |
| **Total Polyphenolics** 225mg | | |
| Catechins 20mg | | |
| Lignins 52mg | | |
| Hydroxycinnamates 8mg | | |
| Other Polyphenolics 146mg | | |
| **Protein** 4g | | |

Figure 2. Sample food content label indicating polyphenolics.

### Conclusion

There should be an attempt to describe phenolic content to consumers in an usable form. Label information based on flavonoid class is the most logical and flexible, especially if total phenol is included, as that accounts for a large quantity of poorly defined phenolic substances. This would provide consumers with far better information than currently available and provide proper incentives to processors to retain these valuable substances in their products.

### References

Bonvehi JS, Coll FV. 2002. Factors affecting the formation of alkylpyrazines during roasting treatment in natural and alkalinized cocoa powder. J Agric Food Chem 50:3743–3750.

Hollman PCH, De Vries JHM, Van Leeuwen SD, Mengelers MJB, Katan MB. 1995. Absorption of dietary quercetin glycosides and quercetin in healthy ileostomy volunteers. Am J Clin Nutr 62:1276–1282.

Jang M, Cai L, Udeani GO, et al. 1997. Cancer chemopreventive activity of resveratrol, a natural product derived from grapes. Science 275:218–220.

Kennedy JA, Taylor AW. 2003. Analysis of proanthocyanidins by high-performance gel permeation chromatography. J Chromatography A 995:99–107.

Kennedy JA, Matthews MA, Waterhouse AL. 2000. Changes in grape seed polyphenols during fruit ripening. Phytochemistry 55:77–85.

Kennedy JA, Jones GP. 2001. Analysis of proanthocyanidin cleavage products following acid-catalysis in the presence of excess phloroglucinol. J Agric Food Chem 49:1740–1746.

Lea AGH. 1978. The phenolics of ciders: Oligomeric and polymeric procyanidins. J Sci Food Agric 29:471–477.

Liao S, Umekita Y, Guo J, Kokontis JM, Hiipakka RA. 1995. Growth inhibition and regression of human prostate and breast tumors in athymic mice by tea epigallocatechin gallate. Cancer Lett 96:239–243.

Lodovici M, Casalini C, De Filippo C, et al. 2000. Inhibition of 1,2-dimethylhydrazine-induced oxidative DNA damage in rat colon mucosa by black tea complex polyphenols. Food Chem Toxicol 38:1085–1088.

Price SF, Breen PJ, Valladao M, Watson BT. 1995. Cluster sun exposure and quercetin in Pinot noir grapes and wine. Am J Enol Vitic 46:187–194.

Rieger T. 2000. Micro-oxygenation presents promise with potential peril for quality winemaking. Vineyard and Winery Management. p 83–86.

Rigaud J, Escribano-Bailon MT, Prieur C, Souquet JM, Cheynier V. 1993. Normal-phase high-performance liquid chromatographic separation of procyanidins from cacao beans and grape seeds. J Chromatography A 654:255–260.

Ritchey JG, Waterhouse AL. 1999. A standard red wine: Monomeric phenolic analysis of commercial Cabernet Sauvignon wines. Am J Enol Vitic 50:91–100.

Waterhouse AL, Kennedy JA. 2004. Revealing the mysteries of red wine color. Washington, DC: American Chemical Society, in press.

Wiseman S, Waterhouse A, Korver O. 2001. The health effects of tea and tea components: opportunities for standardizing research methods. Crit Rev Food Sci Nutr 41(suppl.):387–412.

